# Fractal Structure and Non-Extensive Statistics

**DOI:** 10.3390/e20090633

**Published:** 2018-08-24

**Authors:** Airton Deppman, Tobias Frederico, Eugenio Megías, Debora P. Menezes

**Affiliations:** 1 Instituto de Física, Universidade de São Paulo, Rua do Matão Travessa R Nr.187, Cidade Universitária, CEP 05508-090 São Paulo, Brazil; 2 Instituto Tecnológico da Aeronáutica, 12228-900 São José dos Campos, Brazil; 3 Departamento de Física Teórica, Universidad del País Vasco UPV/EHU, Apartado 644, 48080 Bilbao, Spain; 4 Departamento de Física Atómica, Molecular y Nuclear and Instituto Carlos I de Física Teórica y Computacional, Universidad de Granada, Avenida de Fuente Nueva s/n, 18071 Granada, Spain; 5 Departamento de Física, CFM, Universidade Federal de Santa Catarina, CP 476, CEP 88040-900 Florianópolis, Brazil

**Keywords:** fractal structure, non-extensive statistics, Tsallis statistics, self-similarity, scale invariance

## Abstract

The role played by non-extensive thermodynamics in physical systems has been under intense debate for the last decades. With many applications in several areas, the Tsallis statistics have been discussed in detail in many works and triggered an interesting discussion on the most deep meaning of entropy and its role in complex systems. Some possible mechanisms that could give rise to non-extensive statistics have been formulated over the last several years, in particular a fractal structure in thermodynamic functions was recently proposed as a possible origin for non-extensive statistics in physical systems. In the present work, we investigate the properties of such fractal thermodynamical system and propose a diagrammatic method for calculations of relevant quantities related to such a system. It is shown that a system with the fractal structure described here presents temperature fluctuation following an Euler Gamma Function, in accordance with previous works that provided evidence of the connections between those fluctuations and Tsallis statistics. Finally, the scale invariance of the fractal thermodynamical system is discussed in terms of the Callan–Symanzik equation.

## 1. Introduction

As the formulation of new mathematical tools opens opportunities to describe systems of increasing complexity, entropy emerges as an important quantity in different areas. In recent years, our knowledge about the role played by entropy in physics as well as in other fields have increased rapidly in part, at least, due to the formulation of new entropic forms that generalize in some way the one first proposed by Boltzmann. The non additive entropy, $S_{q}$, introduced by Tsallis [1] has found wide applicability, triggering interesting studies on the deepest meaning of entropy and on its importance in the description of complex systems [2,3,4,5,6,7].

The full understanding of the non-extensive statistics formulated by Tsallis, however, has not been accomplished yet. Four different connections between Boltzmann and Tsallis statistics have been proposed so far [8,9,10,11,12,13], all of them giving a clear meaning to the entropic index, *q* that appears in the non-extensive case and, in all connections, Boltzmann statistics are obtained as a special case. Nevertheless, it seems that the physical meaning of this parameter is not understood in the general case, and the difficulty to grasp the significance of the entropic index may be related to the fact that this quantity never appeared before in thermodynamics, while temperature, even if it appears as another parameter in statistical mechanics, had already an intuitive meaning in the description of thermodynamical systems. This fact, however, cannot diminish the importance of the index *q* in the formulation and description of systems where Boltzmann statistics is not suitable.

In the present work, we make a detailed analysis of the fourth of those connections, where a system featuring fractal structure in its thermodynamic properties, which was named thermofractals [12], has been shown to follow Tsallis statistics. These fractals are relatively simple systems: they are conceived as objects with an internal structure that can be considered as an ideal gas of a specific number of subsystems, which, in turn, are also fractals of the same kind. The self-similarity between fractals at different levels of the internal structure follows from its definition and reveals the typical scale invariance. It has been shown that thermodynamical systems with the structure studied in the present work show fractional dimensions [12], another feature shared with fractals in general. The fractal dimension can be related to the fact that the system energy is proportional to a power of the number of particles, this power being different from unit. This and other aspects of those systems will be discussed in the present work.

Although the motivation that prompted the formulation of thermofractals was related to applications of Tsallis distributions in high energy physics [14,15,16,17,18,19,20,21,22,23,24,25,26,27,28,29,30], Hadron physics [28,31,32,33,34,35], astrophysics [32,33,34,36] and cosmic ray spectrum [37], the concept of thermofractals is, in fact, general and, in principle, could find applications in other fields. Being a way to relate formally Tsallis and Boltzmann statistics, the analysis of these fractals may shed some light on the open questions about the meaning of the entropic parameter and on the fundamental basis of the non-extensive statistics, and, in this way, it can also contribute to a better understanding of entropy. In this regard, it is worth mentioning that fractals was one of the starting points for the formulation of the generalized statistics [1]. In spite of our objective here being the study of the general properties of thermodynamical fractals, the results obtained in the present work offer a new perspective in the analysis of Hadron structure. This new perspective will be exploited in a future paper.

This work is organized as follows: in Section 2, the main aspects of thermodynamical fractals are reviewed; in Section 3, the fractal structure is analyzed in detail; in Section 4, a diagrammatic scheme to facilitate calculations is introduced, and some examples are given; in Section 5, it is shown that the temperature of the fractal system addressed here fluctuates according to the Euler Gamma Function, a kind of temperature fluctuation already associated with Tsallis statistics; in Section 6, we analyse the scale invariance of thermofractals in terms of the Callan–Symanzik equation, a result that may be of importance for applications in Hadron physics; in Section 7, our conclusions are presented.

## 2. Fractals and Tsallis Statistics

From a mathematical point of view, the basic difference between Boltzmann and Tsallis statistics is the probability factor, P(E), which is an exponential function of energy in the case of Boltzmann statistics, and in the non-extensive statistics proposed by Tsallis is a function called q-exponential, given by

(1) $$P\left(\varepsilon \right)=A{\left[1+\left(q-1\right)\frac{\varepsilon }{k\tau }\right]}^{-1/(q-1)}$$

where τ is associated with the temperature, *k* is the Boltzmann constant, *A* is a normalization constant and *q* is the so-called entropic factor, which is a measure of the deviation of the system thermodynamical behavior from the one predicted by the extensive statistics.

The emergence of the non-extensive behavior has been attributed to different causes: long-range interaction, correlations, memory effects, which would lead to a special class of Fokker–Planck equation that would lead to a non-extensive behavior [8], temperature fluctuation [9,10], and finite size of the system [11]. In this work, we analyze in detail a thermodynamical system recently proposed that presents a fractal structure in its thermodynamical functions, which leads to a natural description of its properties in terms of Tsallis statistics [12], and we show that such a system presents a fractal structure in its momentum space. Three important properties for systems with fractal structure are defined in [12,38] and will be used in the present work:

1. It presents a complex structure with a number $N^{\prime }$ of compound systems that present the same properties as the parent system.
2. The internal energy, *E*, and the kinetic energy, *F*, of each compound system are such that the ratio E/F follows a distribution $\tilde{P}\left(\varepsilon \right)$.
3. At some level of the internal struture, the fluctuations of internal level of the compund systems are small enough to be disconsidered, and then their internl energy can be regarded as constant.

In the study of the thermodynamical properties of the fractal system of interest here, an important quantity is the partition function, defined as

$$Z=\int_{0}^{\infty }\rho \left(U\right)exp\left(-\frac{U}{kT}\right)dU$$

where ρ(U) is the density of states. The probability of finding the system at an energy between *U* and U+dU is, accordingly, given by

$$P\left(U\right)dU=\frac{\rho (U)exp(-U/kT)}{Z}dU$$

For simplicity, here we will use the quantity

(2) $$\Omega =\int_{0}^{\infty }P\left(U\right)dU$$

which is, obviously, identical to unit.

The main characteristic of the fractal system [12] of interest here is that Ω, which can be written in Boltzmann statistics as

(3) $$\Omega =\int_{0}^{\infty }A\rho \left(U\right)exp\left(-\frac{U}{kT}\right)dU$$

where $A=Z^{-1}$. ρ(U) is a particular density of states characteristic of such fractal system, results in being equivalent to the integration over all possible energies of the q-exponential function, that is,

(4) $$\Omega =\int_{0}^{\infty }A{\left[1+\left(q-1\right)\frac{\varepsilon }{k\tau }\right]}^{-1/(q-1)}d\varepsilon$$

This result shows, therefore, that, for systems with a particular density of states, will be presented in the following: Tsallis statistics can substitute Boltzmann statistics while all the details of the internal structure of the system are ignored. In particular, this system presents a fractal structure in some thermodynamical quantities, and, consequently, it shows an internal structure with self-similarity, i.e., the internal components are identical to the main system after rescaling.

The importance of this result is two-fold: in one hand, it allows for understanding the emergence of non-extensivity and the applicability of Tsallis entropy becomes clear, with the entropic index, *q*, being given by quantities well defined in the Boltzmann statistics; on the other hand, the structure obtained resembles in many ways strongly interacting systems, where Tsallis statistics has been used, indeed, to describe experimental distributions [25,39,40,41].

The particular fractal structure that leads to Tsallis statistics has a density of states given by

(5) $$\rho \left(\varepsilon ,F\right)=A^{\prime }F^{\frac{3N^{\prime }}{2}-1}{\left[\tilde{P}\left(\varepsilon \right)\right]}^{\nu }$$

where *F* and ε are independent quantities and $A^{\prime }=AkT$. The remaining part of the total energy, E=U−F, is such that

(6) $$\frac{\varepsilon }{kT}=\frac{E}{F}$$

The exponent ν in Equation (5) is a constant that will be related, in the following, to the entropic index, and $\tilde{P}\left(\varepsilon \right)$ to the Tsallis distribution. Notice that the phase space corresponding to a variation dU is given, in terms of the new variable, by dU=dFdε, since the two variables are independent.

Substituting Equation (5) in Equation (3), it follows that

(7) $$\Omega =\int_{0}^{\infty }\int_{0}^{\infty }AF^{\frac{3N}{2}-1}exp\left\{-\frac{\alpha F}{kT}\right\}dF{\left[\tilde{P}\left(\varepsilon \right)\right]}^{\nu }d\varepsilon$$

with $N=N^{\prime }+2/3$ and α=1+ε/kT. Observe that now we have integrations on the independent variables *F* and ε. It will be clear in the next section that the integration in *F* is equivalent to an integration on the compound system momentum, and that the integration on ε is related to an integration over the energy of a given component of the system, namely, its subsystems.

It is straightforward to verify that Ω reduces to Equation (1) if $\tilde{P}\left(\varepsilon \right)$ is itself a q-exponential. In fact, defining

(8) $$\tilde{P}\left(\varepsilon \right)={\left(1+\frac{\varepsilon }{NkT}\right)}^{-\frac{3N\nu }{2(1-\nu )}}$$

substituting Equation (5) into Equation (3) and integrating the last equation in *F*, it will result in Equation (4) when the following substitutions are made:

(9) $$\left\{\begin{array}{c} q-1=\frac{2}{3N}\left(1-\nu \right),\\ T=\frac{\tau }{N(q-1)} \end{array}\right.$$

With these substitutions, the density distribution results in being

(10) $$\tilde{P}\left(\varepsilon \right)={\left[1+\left(q-1\right)\frac{\varepsilon }{k\tau }\right]}^{-\frac{1}{q-1}}$$

Comparing Equations (2) and (4), one can see that the energy distribution of the system, P(U) is equal to the probability density $\tilde{P}\left(\varepsilon \right)$. Hence, the energy distribution of the system follows the same distribution of the energy distribution of the compound system internal energy, i.e.,

(11) $$P\left(U\right)∼\tilde{P}\left(\varepsilon \right)$$

This result shows that some properties of the main system are found also in its compound systems, a self-similarity property that is present in the system with a fractal structure. In fact, the system described by the density of states given by Equation (5) is a fractal [12], and below its structure is discussed in detail. Moreover, the distribution given by Equation (10) is the well-known Tsallis distribution, hence we can conclude that using Tsallis statistics all complexity of the fractal system is taken into account in a rather simple way, since, from the non-extensive entropy associated with these statistics, all thermodynamics properties can be derived by the usual thermodynamic relations [42,43].

## 3. Fractal Structure

The results obtained in the last section show that the system with the density of states given by Equation (5) presents self-similarity, allowing one to interpret it as a fractal system. In this section, such structure will be analyzed, and it will be shown that such a system is a fractal in the energy-momentum space. Notice that Equation (7) can be written as

(12) $$\Omega =\int_{0}^{\infty }\int_{0}^{\infty }\left(AkT\right)F^{\frac{3N^{\prime }}{2}-1}exp\left(-\frac{F}{kT}\right)dFexp\left(-\frac{E}{kT}\right)dE$$

The most evident aspect of a fractal structure is its scale invariance. For the system studied here, it means not only that the self-consistency relation represented by Equation (11) must be valid, but also that for the kinetic energy, *F*, the distributions must be the same at all levels of the fractal structure. From Equation (5), it follows that the distribution for *F* is

(13) $$\omega \left(F\right)=\int_{0}^{\infty }A^{\prime }F^{\frac{3N}{2}-1}\exp\left(-\frac{F}{kT}\right)dF$$

which represents a Maxwellian distribution of energy. Therefore, the scale invariance of thermofractals will be accomplished with the requirement that the kinetic energy distribution and the internal energy distribution are invariant under a scale transformation, so

(14) $$\frac{F^{\left(0\right)}}{T^{\left(0\right)}}=\frac{F^{\left(n\right)}}{T^{\left(n\right)}}$$

and

(15) $$\frac{\varepsilon }{kT}=\frac{E^{\left(n\right)}}{F^{\left(n\right)}}$$

remains constant, hence

(16) $$\frac{E^{\left(0\right)}}{T^{\left(0\right)}}=\frac{E^{\left(n\right)}}{T^{\left(n\right)}}$$

Here, and in what follows, we use upper index (0) to refer to quantities for the initial level of the thermofractal structure, or main system, and upper index (n) to refer to quantities for the *n*-th level of the structure. The energy of the initial thermofractal, or main system, is $E=E^{\left(0\right)}$, and the temperature of the internal structure to this level is $T=T^{\left(1\right)}$.

It is interesting to express the scaling properties in terms of the fractal dimension, which is one of the distinguishing properties of fractals and expresses the fact that some quantities do not scale as one could naively expect from the topological dimension of the system. In the present case, as it was shown in Reference [12], energy and particle multiplicity do not increase in the same way, a different behavior from that found in an extensive ideal gas. In fact, in [12], the subsystem energies obey a geometric ratio given by:

(17) $$\lambda_{n}=\frac{E^{\left(n\right)}}{E^{\left(0\right)}}={\left(\frac{1}{N}\right)}^{\frac{n}{1-D}}$$

where

(18) $$D=1+\frac{\log N^{\prime }}{\log R}$$

is the fractal dimension. Here, *R* is the ratio between the internal energy of a subsystem and that of its parent system, and is given in terms of the parameters *q* and *N* by

(19) $$R=\frac{\left(q-1\right)N/N^{\prime }}{3-2q+\left(q-1\right)N^{\prime }}$$

The internal energy distribution scales by a factor

(20) $$\lambda_{n}=\frac{E^{\left(n\right)}}{E^{\left(0\right)}}=\frac{T^{\left(n\right)}}{T^{\left(0\right)}}={\left(\lambda \right)}^{n}$$

defining the quantity $\lambda =1/N^{\frac{1}{1-D}}$, and

(21) $$T^{\left(n\right)}={\left(\frac{1}{N}\right)}^{\frac{n}{1-D}}T$$

Therefore, fractals with different internal energies present energy distributions that are similar and scales with the internal energy of the subsystems, that is,

(22) $$P\left(E^{\left(n\right)}\right)dE^{\left(n\right)}=\lambda_{n}P\left(E^{\left(0\right)}\right)dE^{\left(0\right)}$$

Remarkably, as all energies are rescaled, it also happens ε to be rescaled; therefore, one has

(23) $$\frac{\varepsilon }{k\tau }=\frac{\varepsilon^{\left(n\right)}}{k\tau^{\left(n\right)}}$$

with $\tau^{\left(n\right)}$ determined by Equations (9) and (21). Thus, the argument of the *q*-exponential function in the probability distribution $P\left(\varepsilon \right)=Ae_{q}\left(-\varepsilon /\left(k\tau \right)\right)=A\tilde{P}\left(\varepsilon \right)$ does not change when we move from one level of the system to its next level. This is, in fact, the essence of self-similarity, and P(ε) is the self-similar distribution. Another interesting feature is that

(24) $$A^{\left(n\right)}d\varepsilon^{\left(n\right)}=Ad\varepsilon$$

In what follows, the structure of the system and its subsystem just described will be investigated in detail. For the sake of clarity, the symbols

(25) $$\tilde{P}\left(\varepsilon \right)=\frac{P(\varepsilon )}{A}=e_{q}\left(-\varepsilon /\left(k\tau \right)\right)$$

will be used. Note that $\tilde{P}\left(\varepsilon \right)Ad\varepsilon$ is dimensionless. Due to property 2 of thermofractals, one has at the level n−1 of the fractal structure

(26) $$A^{\left(n\right)}dE^{\left(n\right)}={\left[\tilde{P}\left(\varepsilon \right)\right]}^{\nu }Ad\varepsilon \;\left(\frac{F^{\left(n\right)}}{kT^{\left(n\right)}}\right)$$

where $F^{\left(n\right)}$ is the total kinetic energy of the compound fractals and $E^{\left(n\right)}=F^{\left(n\right)}\varepsilon^{\left(n\right)}/kT^{\left(n\right)}$ is their total internal energy. The following normalized energies will be adopted:

(27) $$f^{\left(n\right)}=\frac{F^{\left(n\right)}}{kT^{\left(n\right)}}$$

and

(28) $$\epsilon^{\left(n\right)}=\frac{E^{\left(n\right)}}{kT^{\left(n\right)}}$$

with n=1,2,… corresponding to the level of the fractal structure. Note that the normalized energies are dimensionless and scale invariant.

Given a fractal with non-extensive temperature τ, the subsystem energy, $\varepsilon^{\left(n\right)}$, fluctuates according to the distribution

(29) $$P\left(\varepsilon^{\left(n-1\right)}\right)d\varepsilon^{\left(n-1\right)}={\left[1+\left(q-1\right)\frac{\varepsilon }{k\tau }\right]}^{-\frac{1}{q-1}}Ad\varepsilon$$

and, generalizing Equation (7) to any subsystem level n−1, one can write (see Equation (A10)) in the Appendix A):

(30) $$\Omega_{n}=\frac{A}{\Gamma (3N^{\prime }/2)}\int_{0}^{\infty }\int_{0}^{\infty }{\left(f^{\left(n-1\right)}\right)}^{\frac{3N^{\prime }}{2}-1}e^{-\alpha f^{\left(n-1\right)}}{\left[\tilde{P}\left(\varepsilon \right)\right]}^{\nu }f^{\left(n-1\right)}d\varepsilon df^{\left(n-1\right)}$$

$\Omega_{n}$ represents the energy distribution of a constituent fractal at the *n*-th subsystem level of the main system.

Let $f_{i}^{\left(n\right)}$ correspond to the kinetic energy of the *i*-th constituent fractal at the *n*-th level of the fractal subsystem structure, each one having an internal energy determined by $\epsilon^{\left(n\right)}=\varepsilon f_{i}^{\left(n\right)}$. Equation (30) can be written in terms of the kinetic and internal energy of each constituent subsystem fractal, since

(31) $$\int_{0}^{\infty }\int_{0}^{\infty }{\left[f^{\left(n-1\right)}\right]}^{(3N^{\prime }/2)-1}e^{-f^{\left(n-1\right)}}e^{-\epsilon^{\left(n-1\right)}}{\left[\tilde{P}\left(\varepsilon \right)\right]}^{\nu }Ad\varepsilon \;f^{\left(n-1\right)}df^{\left(n-1\right)}$$

Note that

(32) $$Ad\varepsilon \;f^{\left(n-1\right)}=Ad\varepsilon \frac{F^{\left(n-1\right)}}{kT^{\left(n-1\right)}}=A\lambda_{n}^{-1}\frac{d\varepsilon }{kT}F^{\left(n-1\right)}$$

Therefore, the constant *A* also scales as

(33) $$A^{n}=A\lambda_{n}^{-1}$$

with $A^{0}=A$ being the constant for the main system. This result is consistent with the temperature scale in Equation (21) and with the energy scaling relation in Equation (22). It results that

(34) $$Ad\varepsilon f^{\left(n-1\right)}=A_{n-1}d\epsilon^{\left(n-1\right)}$$

with $\epsilon^{\left(n-1\right)}$ the normalized total internal energy of the thermofractals at the level n−1. Of course,

(35) $$\epsilon^{\left(n-1\right)}=\sum_{i=1}^{N^{\prime }}\epsilon^{\left(n\right)}$$

The term $d\epsilon^{\left(n-1\right)}$ can be written in terms of $d\epsilon^{\left(n\right)}$ as

(36) $$d\epsilon^{\left(n-1\right)}\to \left[\prod_{i=1}^{N^{\prime }}\int_{0}^{\infty }d\epsilon_{i}^{\left(n\right)}\delta \left(\epsilon^{\left(n-1\right)}-\sum_{j=1}^{N^{\prime }}\epsilon_{j}^{\left(n\right)}\right)\right]d\epsilon^{\left(n-1\right)}$$

since it is related to the number of possible states $\left\{\epsilon_{i}^{\left(n\right)}\right\}$ that would sum up the total energy $\epsilon^{\left(n-1\right)}$. The delta function here indicates that $\epsilon^{\left(n-1\right)}$ is equal to the sum of the energies $\epsilon_{j}^{\left(n\right)}$, which is to be found in the interval between $\epsilon^{\left(n-1\right)}$ and $\epsilon^{\left(n-1\right)}+d\epsilon^{\left(n-1\right)}$.

With these definitions, one has

(37) $$\begin{array}{c} P\left(\epsilon^{\left(n-1\right)},f^{\left(n-1\right)}\right)d\epsilon^{\left(n-1\right)}df^{\left(n-1\right)}={\left[f^{\left(n-1\right)}\right]}^{(3N^{\prime }/2)-1}e^{-f^{\left(n-1\right)}}e^{-\epsilon^{\left(n-1\right)}}{\left[\tilde{P}\left(\varepsilon \right)\right]}^{\nu }Ad\varepsilon \;f^{\left(n-1\right)}df^{\left(n-1\right)}=\\ {\left[f^{\left(n-1\right)}\right]}^{(3N^{\prime }/2)-1}e^{-f^{\left(n-1\right)}}e^{-\epsilon^{\left(n-1\right)}}A^{\left(n-1\right)}d\epsilon^{\left(n-1\right)}df^{\left(n-1\right)}=\\ \frac{\Gamma (3N^{\prime }/2)}{{\left[\Gamma \left(3/2\right)\right]}^{N^{\prime }}}\left[\prod_{i=1}^{N^{\prime }}\int_{0}^{\infty }\left(A^{\left(n\right)}kT^{\left(n\right)}\right)d\epsilon_{i}^{\left(n\right)}\int_{0}^{\infty }df_{i}^{\left(n\right)}{\left[f_{i}^{\left(n\right)}\right]}^{\frac{3}{2}-1}e^{-f_{i}^{\left(n\right)}}e^{-\epsilon_{i}^{\left(n\right)}}\delta_{f,n}\delta_{\epsilon ,n}\right]d\epsilon^{\left(n-1\right)}df^{\left(n-1\right)} \end{array}$$

where

(38) $$\delta_{\epsilon ,n}=\delta \left(\epsilon^{\left(n-1\right)}-\sum_{i}\epsilon_{i}^{\left(n\right)}\right)$$

and

(39) $$\delta_{f,n}=\delta \left(f^{\left(n-1\right)}-\sum_{i}f_{i}^{\left(n\right)}\right)$$

Observe that the integrations inside brakets are performed on the variables corresponding to the subsystem level *n*.

In Equation (37), relation (32) was used for writing $d\epsilon_{i}$ in place of dε since

(40) $$f_{i}^{\left(n\right)}=\frac{{\left[p_{i}^{\left(n\right)}\right]}^{2}}{2m_{i}^{\left(n\right)}kT^{\left(n\right)}}$$

with $m_{i}^{\left(n\right)}$ being the mass of the *i*-th constituent fractal. One can identify the mass with the internal energy of the fractal subsystem, so that $m_{i}^{\left(n\right)}=\epsilon^{\left(n\right)}kT^{\left(n\right)}$, following that

(41) $$f_{i}^{\left(n\right)}=\frac{{\left[\pi_{i}^{\left(n\right)}\right]}^{2}}{2\epsilon_{i}^{\left(n\right)}}$$

where

(42) $$\pi_{i}^{\left(n\right)}=\left[p_{i}^{\left(n\right)}/kT^{\left(n\right)}\right]$$

Then, Equation (37) results in (see Appendix A, Equations (A1) and (A10))

(43) $$P\left(f^{\left(n-1\right)},\epsilon^{\left(n-1\right)}\right)=\prod_{i=1}^{N^{\prime }}\left(A^{\left(n\right)}kT^{\left(n\right)}\right)\int_{0}^{\infty }d\epsilon_{i}^{\left(n\right)}\int_{-\infty }^{\infty }d^{3}\pi_{i}^{\left(n\right)}{\left(2\pi \epsilon_{i}^{\left(n\right)}\right)}^{-3/2}e^{-u_{i}^{\left(n\right)}}\delta_{f,n}\delta_{\epsilon ,n}$$

where $u_{i}^{\left(n\right)}=f_{i}^{\left(n\right)}+\epsilon_{i}^{\left(n\right)}$. In Equation (43), the potential Ω is described entirely in terms of the characteristics of the $N^{\prime }$ compound thermofractals at the *n*-th level of the subsystem fractal structure, with $f_{i}^{\left(n\right)}$ and $\epsilon_{i}^{\left(n\right)}$ being related to their kinetic and internal energies, respectively. However,

(44) $$A^{\left(n\right)}kT^{\left(n\right)}=AkT=\left(2-q\right)/\left[N\left(q-1\right)\right]$$

$\epsilon_{i}^{\left(n\right)}=\epsilon_{i}$ and $\pi_{i}^{\left(n\right)}=\pi_{i}$ are independent of *n*, so it results

(45) $$P\left(f^{\left(n-1\right)},\epsilon^{\left(n-1\right)}\right)=\prod_{i=1}^{N^{\prime }}\frac{2-q}{N(q-1)}\int_{0}^{\infty }d\epsilon_{i}\int_{-\infty }^{\infty }d^{3}\pi_{i}{\left(2\pi \epsilon_{i}\right)}^{-3/2}e^{-u_{i}}\delta_{f,n}\delta_{\epsilon ,n}$$

The self-similar relation present in the subsystem fractal structure can be more apparent if Equation (45) is written as

(46) $$P\left(f^{\left(n-1\right)},\epsilon^{\left(n-1\right)}\right)=\prod_{i=1}^{N^{\prime }}\int_{0}^{\infty }A^{\left(n\right)}e^{-\epsilon_{i}^{\left(n\right)}}dE_{i}^{\left(n\right)}\int_{-\infty }^{\infty }d^{3}\pi_{i}^{\left(n\right)}{\left(2\pi \epsilon_{i}^{\left(n\right)}\right)}^{-3/2}e^{-f_{i}^{\left(n\right)}}\delta_{f,n}\delta_{\epsilon ,n},$$

where it is possible to recognize in the term $A^{\left(n\right)}dE_{i}^{\left(n\right)}$ the same expression as in Equation (26), which allows the extension of calculations to include quantities of the next subsystem level in the fractal structure, i.e., level n+1, since

(47) $$A^{\left(n\right)}dE_{i}^{\left(n\right)}=A^{\left(n+1\right)}dE_{i}^{\left(n+1\right)}$$

and

(48) $$e^{-\epsilon_{i}^{\left(n\right)}}=e^{-\epsilon_{i}^{\left(n+1\right)}}$$

In addition, due to Equation (7),

(49) $$A^{\left(n+1\right)}e^{-\epsilon_{i}^{\left(n+1\right)}}dE_{i}^{\left(n+1\right)}=A^{\left(n+1\right)}{\left(F^{\left(n+1\right)}\right)}^{\frac{3N}{2}-1}\exp\left(-\frac{\alpha F^{\left(n+1\right)}}{kT^{\left(n+1\right)}}\right)dF^{\left(n+1\right)}{\left[\tilde{P}\left(\varepsilon \right)\right]}^{\nu }d\varepsilon^{\left(n+1\right)}$$

which allows the passage to the next subsystem level by following all the steps described above. Before going into further calculations, however, a diagrammatic description will be introduced.

## 4. Diagrammatic Representation

**Figure 1:**
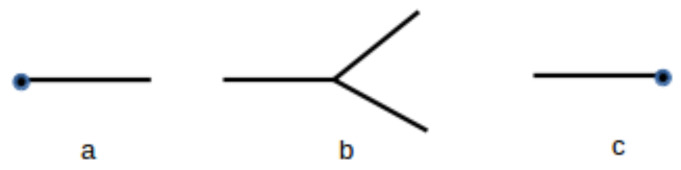
Basic diagrams for the fractal structure: (**a**) main fractal; (**b**) vertex; (**c**) final fractal.

It is possible to have a diagrammatic representation of the probability densities that can facilitate calculations of Ω and other relevant quantities. In Figure 1, the basic diagram symbols are presented, adopting $N^{\prime }=2$ for simplicity. Each of the basic diagrams correspond to a mathematical expression, and the correspondence can be established as follows:

1. A line corresponds to a term
  (50) $$\int_{-\infty }^{\infty }d^{3}\pi \epsilon^{-3/2}e^{-f}\;$$
  with $f=\pi^{2}/\left(2\epsilon \right)$ and ϵ=(u−f), where *u* is the total energy of the fractal represented by the line.
2. A vertex corresponds to the term
  (51) $${\left(2\pi \right)}^{-3/2}\prod_{i=1}^{N^{\prime }}\;\delta \left(f-\sum_{j=1}^{N^{\prime }}f_{j}\right)\;$$
3. To each final line, i.e., those lines that do not finish in a vertex, the associated term reads
  (52) $$\int_{0}^{\infty }A\;kT\;e^{-\epsilon }{\left[\tilde{P}\left(\varepsilon \right)\right]}^{\nu }d\epsilon \;$$

The simplest diagram of interest is a line with a vertex where each branch is a final line. In this case, the diagram scheme results in

(53) $$\begin{array}{c} \int_{-\infty }^{\infty }d^{3}\pi^{\left(n\right)}{\left(\epsilon^{\left(n\right)}\right)}^{-3/2}e^{-f^{\left(n\right)}}{\left(2\pi \right)}^{-3/2}\prod_{i=1}^{N^{\prime }}\;\delta_{f,n}\delta_{\epsilon ,n}\int_{-\infty }^{\infty }d^{3}\pi_{i}^{n+1}{\left(\epsilon_{i}^{\left(n+1\right)}\right)}^{-3/2}e^{-f_{i}^{n+1}}\;\\ \times \int_{0}^{\infty }A\;kT\;e^{-\epsilon_{i}^{n+1}}{\left[\tilde{P}\left(\varepsilon \right)\right]}^{\nu }d\epsilon_{i}^{n+1} \end{array}$$

**Figure 2:**
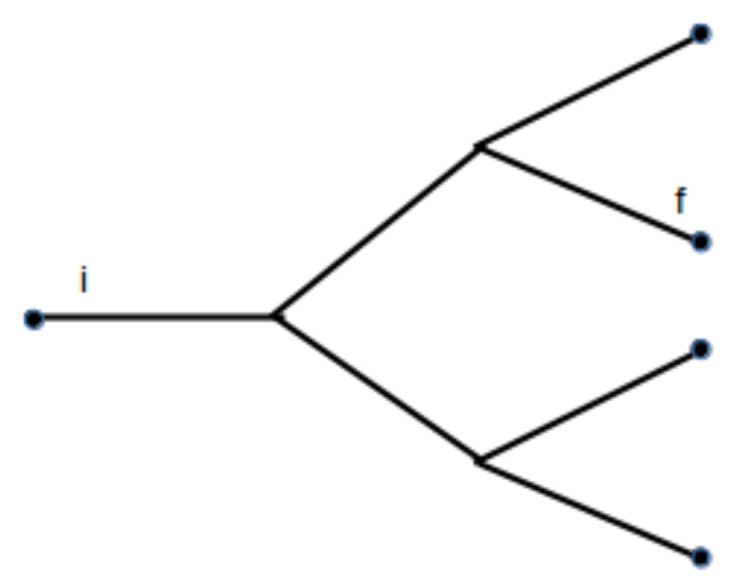
Example of a tree graph representing the different levels of a fractal.

Delta functions can be included to fix energy and momentum of some of the fractals at any level. As an example, consider the graph shown in Figure 2. Observe that there are two levels of the subsystem structure: the initial fractal has well defined momentum (it is indicated by *i*), and, in the second level, one of the subsystems has well defined energy and momentum.

Such a diagram gives the probability to find a constituent subsystem fractal *f* at the third level of the initial fractal *i*. According to the diagrammatic rules, one has

(54) $$P_{i,f}=\prod_{i=1}^{N^{\prime }}\;\delta_{f_{i},1}\int_{-\infty }^{\infty }d^{3}\pi_{i}\epsilon_{i}^{-3/2}e^{-f_{i}}\prod_{j=1}^{N^{\prime }}\;\delta_{f_{i,j},2}\int_{-\infty }^{\infty }d^{3}\pi_{i,j}\epsilon_{i,j}^{-3/2}e^{-f_{i,j}}\times \int_{0}^{\infty }A\;kT\;e^{-\epsilon_{i,j}}{\left[\tilde{P}\left(\varepsilon \right)\right]}^{\nu }d\epsilon_{i,j}\delta_{f_{1,2},f_{f}}$$

where $\delta_{f_{1,2},f_{f}}$ determines the kinetic part of the fractal indicated by *f* at the second level.

It is also possible to consider the subsystem fractal structure in the opposite way: given $N^{\prime }$ fractals with energies $\left\{f_{1},\epsilon_{1},\ldots \;,f_{N^{\prime }},\epsilon_{N^{\prime }}\right\}$ varying in the range $df_{1},f\epsilon_{1},\ldots ,df_{N^{\prime }},d\epsilon_{N^{\prime }}$, the probability that they form a single fractal with energies $f=f_{1}+,\ldots \;,+f_{N^{\prime }}$ and $\epsilon =\epsilon_{1}+,\ldots \;,+\epsilon_{N^{\prime }}$ is given by

(55) $$P\left(E\right)dE=f^{(3N^{\prime }/2)-1}e^{-f}e^{-\epsilon }{\left[\tilde{P}\left(\varepsilon \right)\right]}^{\nu }dfAd\varepsilon$$

with E/kT=f+ϵ and ε=(ϵ/f)kT. This result is a direct consequence of the fact that thermofractals are in thermal equilibrium. After integrating on *f*, one obtains

(56) P(E)dE=P(ε)dε

showing the consistency of the fractal description introduced in the present work.

**Figure 3:**
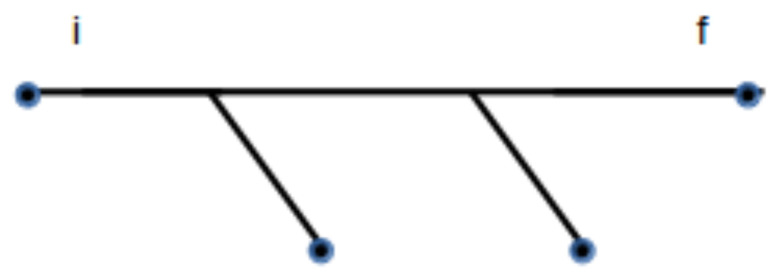
The same diagram of Figure 2 represented as a linear graph. This is possible by rearranging terms in the summation of different contributions and using the merging property of thermofractals.

The process described in Equation (55) corresponds to $N^{\prime }$ fractal subsystems merging into a single one. In the example given above and described in Figure 2, the final system generated from the lower branch at the first level can be merged into a single fractal. The tree diagram can then be reduced to a linear diagram, as shown in Figure 3, resulting in a simpler expression for the probability calculated in that example. In this case, the result is

(57) $$\begin{array}{cc} P_{i,f}= & \prod_{i=1}^{N^{\prime }}\;\delta_{f_{i},1}\delta_{\epsilon_{i},1}\int_{-\infty }^{\infty }d^{3}\pi_{i}\epsilon_{i}^{-3/2}e^{-f_{i}}\prod_{j=1}^{N^{\prime }}\;\delta_{f_{1,j},2}\delta_{\epsilon_{1,j},2}\int_{-\infty }^{\infty }d^{3}\pi_{1,j}\epsilon_{1,j}^{-3/2}e^{-f_{1,j}}\;\times \\ & \int_{0}^{\infty }A\;kT\;e^{-\epsilon_{1,j}}{\left[\tilde{P}\left(\varepsilon \right)\right]}^{\nu }d\epsilon_{1,j}\delta_{\epsilon_{1,2},\epsilon_{f}}\delta_{f_{1,2},f_{f}} \end{array}$$

## 5. Temperature Fluctuation in Thermofractals

On the right-hand side of the last equality in Equation (37), the distribution of the kinetic energy of the thermofractals at the *n*th level is given by

(58) $$P\left(f_{i}^{\left(n\right)}\right)df_{i}^{\left(n\right)}={\left[f_{i}^{\left(n\right)}\right]}^{\frac{3}{2}-1}e^{-f_{i}^{\left(n\right)}}$$

with

(59) $$f_{i}^{\left(n\right)}=\frac{F_{i}^{\left(n\right)}}{T^{\left(n\right)}}$$

where $T^{\left(n\right)}$ is the scaled temperature at the *n*th level of the thermofractal. However, at the n−1 subsystem level, there are $N^{\prime }$ thermofractals, and each of them present different internal energies. One could, therefore, write the temperature $T_{j}^{\left(n\right)}$ associated with the thermofractal *j* at the previous level. Then, Equation (58) can be written as

(60) $$P\left(f_{i,j}^{\left(n\right)}\right)df_{i,j}^{\left(n\right)}={\left[f_{i,j}^{\left(n\right)}\right]}^{\frac{3}{2}-1}e^{-f_{i,j}^{\left(n\right)}}$$

for each thermofractal *i* found inside a thermofractal *j* at level n−1, with

(61) $$f_{i,j}^{\left(n\right)}=\frac{F_{i}^{\left(n\right)}}{T_{j}^{\left(n\right)}}$$

Suppose now that, at the *n*th level, the internal energy fluctuations are already small enough to be disregarded and the internal energy is a constant $m_{i}$. Then, according to the diagrammatic rule 3 of thermofractals, the energy fluctuation of the *j*th thermofractal at the n−1 subsystem level is proportional to the kinetic energy fluctuation, that is,

(62) $$P\left(E_{j}\right)\propto \prod_{i}{\left[f_{i}^{\left(n\right)}\right]}^{\frac{3}{2}-1}e^{-f_{i}^{\left(n\right)}+\mu_{i}}$$

where μ=m/kT. However, the product of Gamma functions above is itself Gamma function, as described in the Appendix, resulting

(63) $$P\left(E_{j}\right)dE_{j}\propto {\left[\frac{F}{kT_{j}^{\left(n\right)}}\right]}^{\frac{3N^{\prime }}{2}-1}exp\left\{-\frac{F}{kT_{j}^{\left(n\right)}}\right\}exp\left\{-\frac{M}{kT_{j}^{\left(n\right)}}\right\}d\left(\frac{F}{kT_{j}^{\left(n\right)}}\right)$$

with $M=\sum m_{i}$. Since the thermofractals at the *n*th subsystem level are being considered as structureless particles, the subsystem at level n−1 can be considered as an ideal gas of particles with masses $m_{i}$. The parent thermofractal at level n−2 is therefore formed by $N^{\prime }$ thermofractals, each one considered as an ideal gas of $N^{\prime }$ particles but at different temperatures $T_{j}$ and with total energy $M_{j}$. The probability density to find a set with total internal energy energy *M* is then

(64) $$P\left(M\right)\propto \int_{0}^{\infty }{\left[\frac{F}{kT_{j}^{\left(n\right)}}\right]}^{\frac{3N^{\prime }}{2}-1}exp\left\{-\frac{F}{kT_{j}^{\left(n\right)}}\right\}exp\left\{-\frac{M}{kT_{j}^{\left(n\right)}}\right\}d\left(\frac{F}{kT_{j}^{\left(n\right)}}\right)$$

If, at this stage, one still disregards the thermofractal subsystem structure, the kinetic energy *F* can only be interpreted as a parameter, while the system energy *M* is the only quantity that keeps some physical meaning, besides the temperature that now fluctuates inside the system. When this step is performed, the equation above is interpreted as a Gamma distribution of the inverse temperature β=1/(kT), that is,

(65) $$P\left(\frac{1}{T}\right)d\left(\frac{1}{T}\right)\propto {\left[\frac{F}{kT}\right]}^{\frac{3N^{\prime }}{2}-1}exp\left\{-\frac{F}{kT}\right\}d\left(\frac{F}{kT}\right)$$

The distribution of temperatures as described by Equation (65) was already considered in connection to Tsallis distribution in a different context [9,10,44]. On the other hand, the possibility of an equilibrated system with temperature fluctuation is rather controversial [45,46,47,48]. In the present work, such fluctuations are well defined in association with the fractal structure of the thermodynamics functions of the system analyzed. Temperature fluctuations arising from a multi scale system were already analyzed in Reference [49].

## 6. Callan–Symanzik Equation for Thermofractals

Due to the evident similarities between Hadron structure and thermofractal structure [13,30,33,36,38] and, due to the possible applications of thermofractals or their consequences in Hadron physics [28,31,33,36], astrophysics [28,32] and high energy physics [16,23,24,30], it is possible to show that the thermofractal description has close connections to quantum field theory as far as scaling properties are concerned. This will be done in a future work [50], but it is convenient to advance some aspects as follows.

The simplest diagrammatic representation of the thermofractal evolution form one level to the next level corresponds to a vertex with an initial system characterized by energy and momentum $\left(\epsilon_{0},{\vec{\pi }}_{0}\right)$, as described by diagram in Figure 1b, at an arbitrary level *n* generating $N^{\prime }$ subsystems with $\left(\epsilon_{i},{\vec{\pi }}_{i}\right)$ such that $\epsilon_{0}=\sum \epsilon_{i}$ and ${\vec{\pi }}_{0}=\sum {\vec{\pi }}_{i}$. Such diagram leads to

(66) $$P_{i,f}\propto N^{\prime n}\prod_{i=1}^{N^{\prime }}{\left(2\pi \epsilon_{i}\right)}^{-3/2}{\left[\tilde{P}\left(\varepsilon_{i}\right)\right]}^{\nu }$$

Here, the passage from one level to the next subsystem one represents only an alternative description of the same system. However, one can consider that the initial thermofractal can break into $N^{\prime }$ pieces, each one being a thermofractal. Let *g* be a coupling constant that gives weight to a transition from one subsystem level to another one, and then one can write

(67) $$\Gamma_{i,j}\propto N^{\prime n}\bar{g}\left(\epsilon_{i}\right)\prod_{i=0}^{N^{\prime }}{\left(2\pi \epsilon_{i}\right)}^{-3/2}$$

and the term

(68) $$\bar{g}=g\prod_{i=0}^{N^{\prime }}{\left[P\left(\varepsilon_{i}\right)\right]}^{\nu }$$

can be considered as an effective coupling constant. $\Gamma_{i,j}$ is, then, understood as a vertex function that is clearly scale free. Vertex functions that are invariant under scale transformation can be described by the Callan–Symanzik equation, which played a fundamental role in the determination of the asymptotic freedom in Yang–Mills theory. A thermofractal version of such equation was already derived in Reference [51], and it will be derived here in a different way.

The thermofractal temperature $T^{\prime }=T^{\left(n\right)}$ works, as seen above, as a scale parameter that determines the fractal structure of the subsystem at a certain level, so one can write the factor $N^{\prime n}$ in terms of the subsystem temperature by using Equation (20), i.e.,

(69) $$N^{n}\propto T^{\prime -(1-D)}$$

Since $N=N^{\prime }+2/3$, for the sake of scaling, it will be assumed $N∼N^{\prime }$, which is a good approximation for *n* sufficiently high. It results that the vertex function is

(70) $$\Gamma_{i,j}\propto {\left(kT^{\prime }\right)}^{-(1-D)}g\prod_{i=1}^{N^{\prime }}{\left(2\pi \frac{E_{i}}{kT^{\prime }}\right)}^{-3/2}{\left[P\left(\varepsilon_{i}\right)\right]}^{\nu }$$

Notice that when the scale transformation on energy and momentum is performed, so that $\vec{\pi }\to \lambda \vec{\pi }$ and ϵ→λϵ, the distribution P(ε) remains unchanged, since E/F is invariant. Therefore, it can be left out of the scale invariance analysis of the vertex function studied here. Taking this aspect into account and introducing $M=kT^{\prime }$ for the sake of simplicity, the scale invariance of the vertex function Γ is expressed by

(71) $$\Gamma \left(\vec{\pi },E^{\prime },M^{\prime }\right)\propto {\left(\frac{M^{\prime }}{M}\right)}^{-(1-D)}\Gamma \left(\vec{\pi },E,M\right)$$

where it made use of the scaling property of thermofractals.

From the above expression, it is straightforward to conclude that

(72) $$\begin{array}{c} E_{i}\frac{\partial \Gamma }{\partial E_{i}}=-\frac{3}{2}\Gamma ,\\ M\frac{\partial \Gamma }{\partial M}=\left(\frac{3N^{\prime }}{2}-\left(1-D\right)\right)\Gamma \end{array}$$

and, with these results, one can write

(73) $$\left[M\frac{\partial }{\partial M}+\sum_{i=1}^{N^{\prime }}E_{i}\frac{\partial }{\partial E_{i}}+d\right]\Gamma =0$$

where d=1−D is the anomalous dimension for thermofractals, a result equivalent to the one obtained in Reference [51].

The fact that thermofractals satisfy the Callan–Symanzik equation indicates that, if it is possible to describe such systems through a field theoretical approach, the Yang–Mills theory is the appropriate framework for it. These results, therefore, sets the grounds for a more fundamental description of thermofractals in terms of gauge field theory, but it will be developed in a future work [50].

## 7. Conclusions

In the present work, the structure of a thermodynamical system presenting fractal structure, recently introduced in [12], is investigated in detail. The fractal structure in thermodynamics has been shown to lead to non-extensive statistics in the form of Tsallis statistics; therefore, this system can shed some light on relevant aspects of the generalized statistics.

The study presented here provides evidence of the consistency of the proposed fractal structure of thermodynamical functions that leads to Tsallis statistics [12]. The diagrammatic representation is a good auxiliary tool for calculations. In the present investigation, the scaling features of thermofractals are made clear, and it is concluded that temperature fluctuates from one subsystem level of the thermofractal structure to the other. It is interesting that temperature fluctuations are pointed out as a possible origin of non-extensive statistics [10]; therefore, one can conjecture that thermofractals will present the same temperature fluctuations necessary to obtain Tsallis statistics, as given in Equation (21).

One of the main results obtained in the present work is given by Equation (33), showing that the normalizing quantity increases as the system is described by means of structures at deeper levels, *n*. This is a consequence of the fact that the systems at deeper levels contribute less to the energy fluctuation of the system. It follows, on the other hand, from the fact that, at deeper levels, thermofractals are less massive, and since the energy fluctuation of thermofractals presents self-similarity, energy fluctuation tends to vanish as *n* increases, as described through Equation (22).

Another interesting result within the context of Hadron production in high energy collisions is the scale parameter $\lambda_{n}$, which appears in Equation (20). If $E^{\left(n\right)}=\Lambda$ and $E^{\left(0\right)}=E$, it is obtained that

(74) nlogN=(1−D)log(E/Λ)

Since $N^{n}={\left(N^{\prime }+3/2\right)}^{n}∼M$, for *n* sufficiently large, with *M* being the particle multiplicity, it follows that

(75) logM=(1−D)log(E/Λ)

Thus, the measurement of particle multiplicity in high energy nuclear collisions gives an easy way to access the associated fractal dimension *D* in practice.

In addition, it is shown that thermofractals, when the internal structure is not considered, can be interpreted as an ideal gas with inverse temperature that fluctuates according to the Euler’s Gamma function. Such temperature distribution was already connected to Tsallis distribution, but here it is obtained as a consequence of the fractal structure of thermodynamics functions.

In summary, a diagrammatic prescription for calculations with the fractal structure is introduced, which can certainly help in calculations involving several subsystem levels of the fractal structure, and some examples are presented. In particular, it is shown that the equivalence between tree diagrams and linear diagrams, a result that simplifies the calculations of the relevant quantities. Temperature fluctuations inside the thermofractal is analyzed, reproducing a well-known distribution already connected to Tsallis distribution. The Callan–Symanzik equation for thermofractal structure was obtained, opening the opportunity to develop a field theoretical approach for thermofractals.

## Appendix A. Useful Formulae

The energy distribution of an ideal gas is given by

(A1) $$\int_{0}^{\infty }P\left(F\right)dF=C\int_{-\infty }^{\infty }d^{3N^{\prime }}p_{i}\exp\left[-\beta \sum_{i=1}^{3N^{\prime }}\frac{p_{i}^{2}}{2m}\right]\;$$

where β=1/(kT) and *C* is the normalization constant. If the momenta of the different particles are independent, then

(A2) $$\int_{0}^{\infty }P\left(F\right)dF=C{\left\{\int_{-\infty }^{\infty }dp_{i}\exp\left[-\beta \frac{p_{i}^{2}}{2m}\right]\right\}}^{3N^{\prime }}\;$$

Since

(A3) $$\int_{-\infty }^{\infty }dp_{i}\exp\left[-\beta \frac{p_{i}^{2}}{2m}\right]={\left(2\pi mkT\right)}^{1/2}\;$$

the normalization constant must be chosen as

(A4) $$C={\left(2\pi mkT\right)}^{-3N^{\prime }/2}\;$$

Notice that the integration in Equation (A1) can be performed in terms of the total momentum $p^{2}=\sum_{i=1}^{3N^{\prime }}p_{i}^{2}$, by considering a hypersphere of dimension $n=3N^{\prime }$. Then,

(A5) $$\int_{0}^{\infty }P\left(F\right)dF=C\int_{0}^{\infty }dp\;S_{n}p^{n-1}\exp\left[-\beta \frac{p^{2}}{2m}\right]\;$$

where

(A6) $$S_{n}=\frac{2\pi^{n/2}}{\Gamma (n/2)}$$

is a surface factor for the *n*-dimensional hypersphere, with Γ being the Euler Gamma Function. However, $F=p^{2}/\left(2m\right)$, then

(A7) $$dF=\frac{p}{m}dp\;$$

hence

(A8) $$\frac{dF}{2F}=\frac{dp}{p}\;$$

Therefore, from Equation (A5), one has

(A9) $$\int_{0}^{\infty }P\left(F\right)dF=CS_{n}\frac{{\left(2m\right)}^{n/2}}{2}\int_{0}^{\infty }\frac{dF}{F}F^{n/2}\exp\left(-\beta F\right)\;$$

Substituting the relations for $S_{n}$, *n* and *C*, it results in

(A10) $$\int_{0}^{\infty }P\left(F\right)dF=\int_{0}^{\infty }\frac{1}{\Gamma (3N^{\prime }/2)}\frac{dF}{kT}{\left(\frac{F}{kT}\right)}^{(3N^{\prime }/2)-1}\exp\left(-\beta F\right)\;$$
